# Sexual Dichromatism of the Damselfly *Calopteryx japonica* Caused by a Melanin-Chitin Multilayer in the Male Wing Veins

**DOI:** 10.1371/journal.pone.0049743

**Published:** 2012-11-20

**Authors:** Doekele G. Stavenga, Hein L. Leertouwer, Takahiko Hariyama, Hans A. De Raedt, Bodo D. Wilts

**Affiliations:** 1 Computational Physics, Zernike Institute for Advanced Materials, University of Groningen, Groningen, The Netherlands; 2 Department of Biology, Hamamatsu University School of Medicine, Handayama, Higashi-ku, Hamamatsu, Japan; The Australian National University, Australia

## Abstract

Mature male *Calopteryx japonica* damselflies have dark-blue wings, due to darkly coloured wing membranes and blue reflecting veins. The membranes contain a high melanin concentration and the veins have a multilayer of melanin and chitin. Female and immature *C. japonica* damselflies have brown wings. We have determined the refractive index of melanin by comparing the differently pigmented wing membranes and applying Jamin-Lebedeff interference microscopy. Together with the previously measured refractive index of chitin the blue, structural colour of the male wing veins could be quantitatively explained by an optical multilayer model. The obtained melanin refractive index data will be useful in optical studies on melanized tissues, especially where melanin is concentrated in layers, thus causing iridescence.

## Introduction

Animal coloration is generally distinguished in pigmentary and structural coloration. Melanin pigment, a key component in human hair, skin and eyes, is equally widespread in birds and insects [1]. Its common function is to absorb light, for protection or camouflage. In insects, melanin generally is diffusely distributed in a matrix of chitin, and then causes a brown or black pigmentary colour, depending on the concentration. However, several insect species have melanin concentrated in nanosized layers in the body cuticle and wings, thus causing striking iridescent, structural colours. Notable examples are the jewel beetles and some damselfly species [2] [3] [4] [5] [6].

The iridescent reflections are determined by the layer distances and the refractive index contrast between the differently melanized layers [7] [8]. Whereas the refractive index of insect chitin has been well established to be around 1.55 [9]–[10], the refractive index of melanin has been much less certain. A round figure of 2.0 has been often used in optical modelling studies (e.g. [7] [11] [12] [13] [14]). Because melanin is a strongly absorbing pigment, the value of the refractive index is a complex number, which is difficult to measure accurately. In a brief abstract on sepia melanin, Kurtz et al. [15] reported for the real part of the refractive index at wavelength *λ* = 633 nm a value 1.655±0.008 and for the imaginary part 0.12±0.07. Similar data were obtained by Noyes et al. [5], who investigated the iridescence of the elytra of a buprestid beetle, *Chrysochroa raja*. They measured the reflectance spectra as a function of angle and polarization, and using anatomical data, they fitted the spectra with free parameter multilayer modelling. Noyes et al. [5] thus concluded that the melanin and chitin layers have real refractive index values 1.68 and 1.55 and imaginary refractive index values 0.03 and 0.14, respectively. The latter means that the chitin layers are highly absorbing, whereas all present evidence indicates that chitin absorbs negligibly and at least much less than melanin. In fact, fitting spectra with free-parameter multilayer modelling can be hazardous, as various parameter sets can all give reasonable fits (for an example, see [16]).

In all studies until recently the refractive index values of melanin and chitin were assumed to be wavelength-independent. This is an obvious oversimplification, as for instance melanin absorption strongly decreases with increasing wavelength, and dispersion (wavelength dependence) is a principal characteristic of refractive indices. Yoshioka and Kinoshita [17] were the first to directly investigate the wavelength dependence of the refractive index of melanin by analysing thin sections of the elytra of the congeneric jewel beetle *Chrysochroa fulgidissima*. They found that the (real part of the) refractive index of the melanin layers decreased from ∼1.79 at 400 nm to ∼1.64 at 700 nm; for the chitin layers the corresponding values were 1.61 and 1.54. These values were derived while assuming that the melanin and chitin layers in the beetle elytra are discrete and well separated. Transmission electron micrographs (TEM) however clearly show that the electron density is graded and hence the beetle elytra contain a gradient index multilayer. Assuming a linear relationship between the electron density in the TEM pictures and melanin absorption, and applying multilayer modelling, Stavenga et al. [6] could straightforwardly explain measured reflectance spectra of the elytra of *C*. *fulgidissima*. The real part of the refractive index value of the layers was thus concluded to oscillate between ∼1.60 and ∼1.73 [6]. In summary, all recently obtained data for the refractive index value of melanin point to quite lower values than the commonly assumed value 2.0.

Anatomy of the wing veins of the male damselfly *Calopteryx japonica* revealed a multilayer very similar to that of the jewel beetle elytra [4]. The male damselfly has strikingly dark-blue wings compared to the female, which has rather brown coloured wings; the immature male and female have light-brown wings [4]. The dark colour of the male wings is clearly the result of a high melanin concentration in the wing membrane, and the wing veins will have a gradient melanin concentration alike the beetle elytra. The blue reflection of the wing veins thus could in principle be modelled in the same heuristic way as done for the beetle elytra, but we decided to more directly determine the refractive index of melanin using a novel method based on Jamin-Lebedeff microscopy (in preparation). The obtained data allow us to quantitatively understand the blue reflections of the wing veins by optical multilayer modelling.

## Materials and Methods

### Damselflies

*Calopteryx japonica* damselflies were captured in Saga prefecture (Japan) near public ponds and creeks. Their wings were photographed, and transmittance and reflectance spectra were measured with an integrating sphere as well as with a microspectrophotometer. The presence of melanin was demonstrated by immersing wings in warm 3% H_2_O_2_ [18]; see Supplementary Figure S1. The refractive index of the wing membranes was determined by Jamin-Lebedeff interference microscopy (see Supplementary Figure S2, S3). As a reference control, we used the transparent wings of a dragonfly, a male Black-tailed Skimmer, *Orthetrum cancellatum*, captured near Groningen, the Netherlands, at a public pond. Both, *Calopteryx japonica* and *Orthetrum cancellatum*, are not endangered or protected and no specific permits were required for the described field studies.

### Transmission Electron Microscopy

For transmission electron microscopy (TEM) of the membrane and veins of the wings standard methods were used. Sections were double-stained with 2% uranyl acetate and 0.4% lead citrate solution for 5 min and 3 min, respectively, and observed with a transmission electron microscope (JEM-1220, JEOL) at 80 kV emission voltage. The multilayer structures in the wing veins revealed by the TEM micrographs were processed as follows. The optical density was determined by taking the decadic logarithm of the pixel values averaged over 5 nm thick layers in 500 nm wide and 1050 nm long adjacent lanes. Subsequently, assuming that the refractive index is proportional to the derived average density, the reflectance of the stack was calculated with a model based on classical multilayer theory for dielectric media [6].

### Integrating Sphere Spectrophotometry

Reflectance and transmittance spectra of mature male and female wings were measured with an integrating sphere (AvaSphere-50-Refl) connected to a fibre optic spectrometer (SD2000, Avantes, Eerbeek, the Netherlands). The light source was a deuterium/halogen lamp (AvaLight-D(H)-S). A white reflectance standard (Spectralon, Labsphere, North Sutton, NH, USA) served as a reference (for detailed methodology, see [16]).

### Microspectrophotometry, Absorption Coefficient and Refractive Index

Transmittance spectra of the wing membrane and reflectance spectra of the wing veins were measured with a microspectrophotometer (MSP), which consisted of a xenon light source, a Leitz Ortholux microscope and a CCD detector array spectrometer (AvaSpec-2048, Avantes, Eerbeek, The Netherlands). The microscope objective was an Olympus 20x, NA 0.46. The absorbance, or optical density, *D*(*λ*), is derived from the transmittance, *T*(*λ*), with 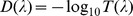, where *λ* is the light wavelength. When the object is homogeneous, its transmittance is
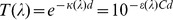
(1)with *κ*(*λ*) the absorption coefficient, *d* the object thickness, *ε*(*λ*) the molar extinction coefficient of the absorbing material, and *C* its concentration. The absorbance then is:



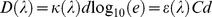
(2)The absorption coefficient is connected with the imaginary part of the complex refractive index, *n* = *n*_R_+*in*_I_, with *n*_R_ and *n*_I_ the real and imaginary parts, by
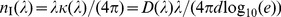
(3)


## Results

### Wing Pigmentation and Melanin Spectra

**Figure 1:**
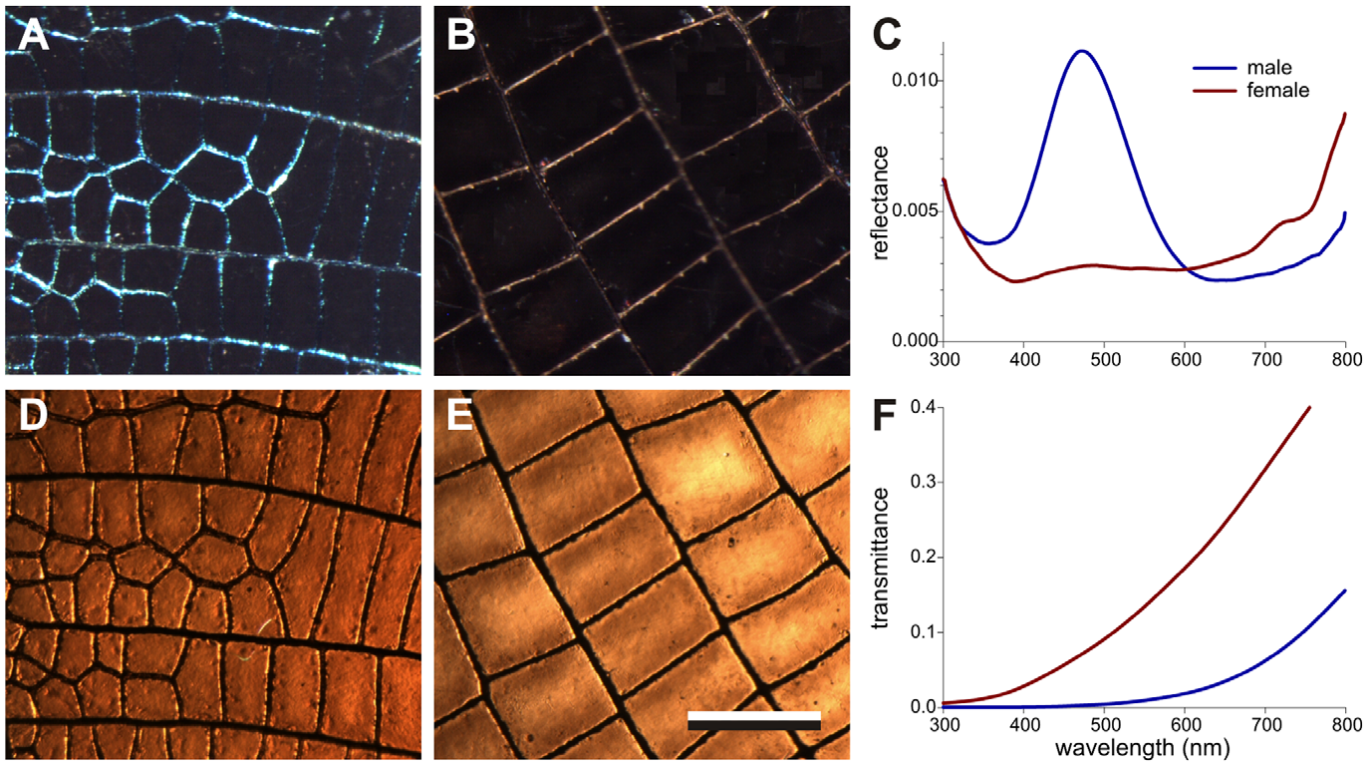
Reflection and transmission of the hindwings of a mature male (A,D) and female (B, E) damselfly *Calopteryx japonica*. **A** The male wing observed with incident light, showing the blue-reflecting veins. **B** The reflection/scattering of the female wing veins is light-brown. **C** Reflectance spectra of the male and female wing measured with an integrating sphere. **D** The male wing (same as **A**) observed with transmitted light. E The female wing (same as **B**) in transmitted light. **F** Transmittance spectra of the male and female wing measured with an integrating sphere. Bar (**A**, **B**, **D**, **E**): 0.5 mm.

Male and female *Calopteryx japonica* damselflies show a distinct dichromatism, that is, have strongly different wing colorations (Figure 1). The wing pattern is determined by the veins, which are joined by thin membranes. When observed with incident illumination, the veins of the male wings reflect in the blue (Figure 1A), but the veins of the female wings are brown (Figure 1B). Reflectance spectra measured with an integrating sphere show a spectrum with a clear, blue peak for the male wing, but the female wing reflectance spectrum is rather indistinct (Figure 1C). Observed with transmitted light, the wing veins stand out as dark lines and the membrane areas are brown. The wing membranes of the male appear more densely pigmented than those of the female (Figure 1D,E). The corresponding transmittance spectra strongly suggest that in both cases the absorbing pigment is melanin (Figure 1F), which was ascertained by immersing the wings in H_2_O_2_, resulting in a severe bleaching (see Supplementary Figure S1).

**Figure 2:**
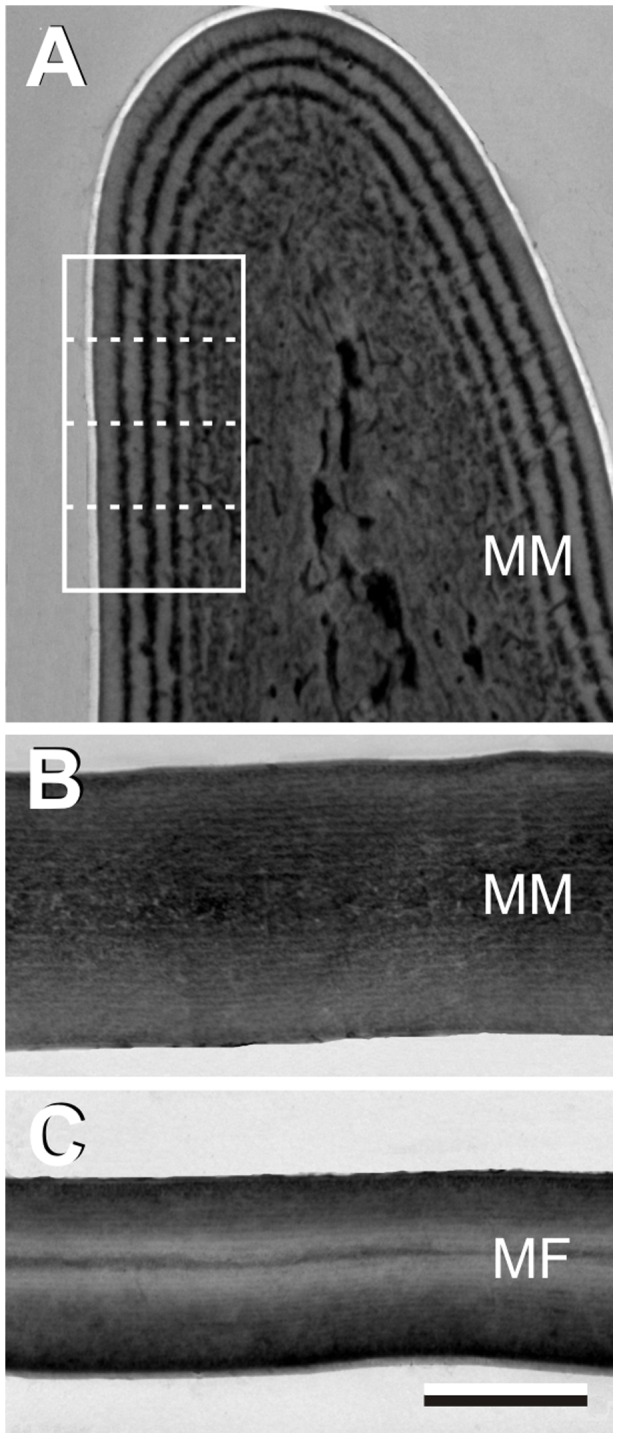
Transmission electron micrographs of wing vein and membrane. A A wing vein of a mature male (MM). The boxed area is used in the calculations of the reflectance spectra of Figure 6. **B** Wing membrane of a mature male. **C** Wing membrane of a mature female (MF). Bar (**A**–**C**): 1 µm.

The blue reflections of the male wing veins have most likely a structural origin. Transmission electron micrographs show a distinct multilayer, with period about 100 nm, parallel with the wing vein surface (Figure 2A), which presumably causes the blue vein coloration. (Transmission electron microscopy of the female wing veins demonstrated that a similar layering does not exist there [4].) The electron micrograph of Figure 2A closely resembles micrographs of other insect cuticle, like that of the jewel beetle [6] [17], and therefore we hypothesized that the multilayer in the wing veins is created by a layered deposition of melanin in a chitin matrix. To be able to quantitatively test this hypothesis, we have to know the refractive index of the melanin. This can in principle be done by Jamin-Lebedeff interference microscopy, but the veins are not well measurable, and therefore we assumed that the melanin in the veins is the same as that contained in the wing membrane. The latter approximates a thin plate (Figure 2B,C), and hence the pigment content in the wing membrane can be favourably assessed. Transmission electron micrographs of the male wing membrane cross-sections show an about homogeneous staining (Figure. 2B). The female wing membrane is slightly layered (Figure 2C), but the layer thicknesses of ∼35 nm and the low density contrasts clearly do not create a structural colour (Figure 1B).

**Figure 3:**
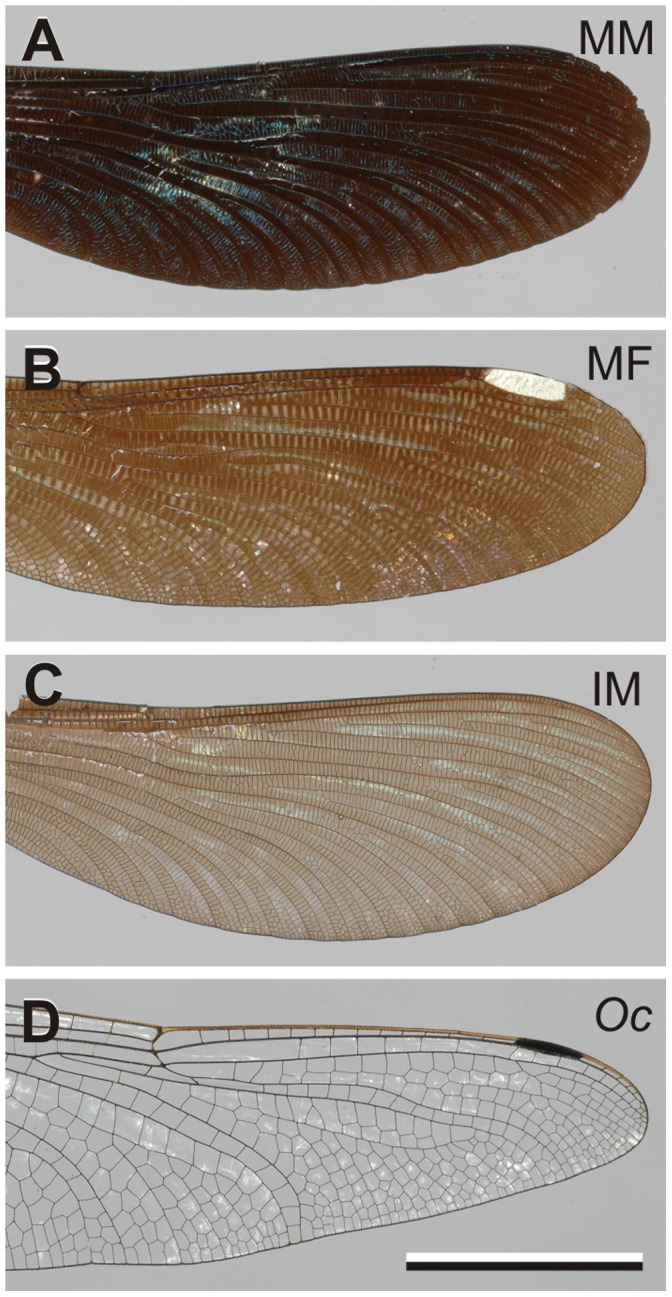
Photographs of the investigated hindwings. **A** Mature male (MM) damselfly, *Calopteryx japonica*. **B** Mature female (MF) damselfly. **C** Immature male (IM) damselfly. **D** Male Black-tailed Skimmer, *Orthetrum cancellatum* (*Oc*). Bar (**A**–**D**): 1 cm.

To estimate how pigment concentration determines coloration, we performed transmission microspectrophotometry on wing membranes of the mature male (MM) damselfly, *Calopteryx japonica*, the mature female (MF), and the immature male (IM); visual inspection immediately reveals their different pigment content (Figure 3A–C). (We have not included data of the immature female here, because it was very similar to the immature male data; see [4].) We have used the Black-tailed Skimmer dragonfly, *Orthetrum cancellatum* (*Oc*), as a reference, because it has very transparent wing membranes (Figure 3D). To avoid reflection and scattering on the membrane surfaces, we measured the transmittance of the wing membranes of wing pieces immersed in refractive index matching fluid.

**Figure 4:**
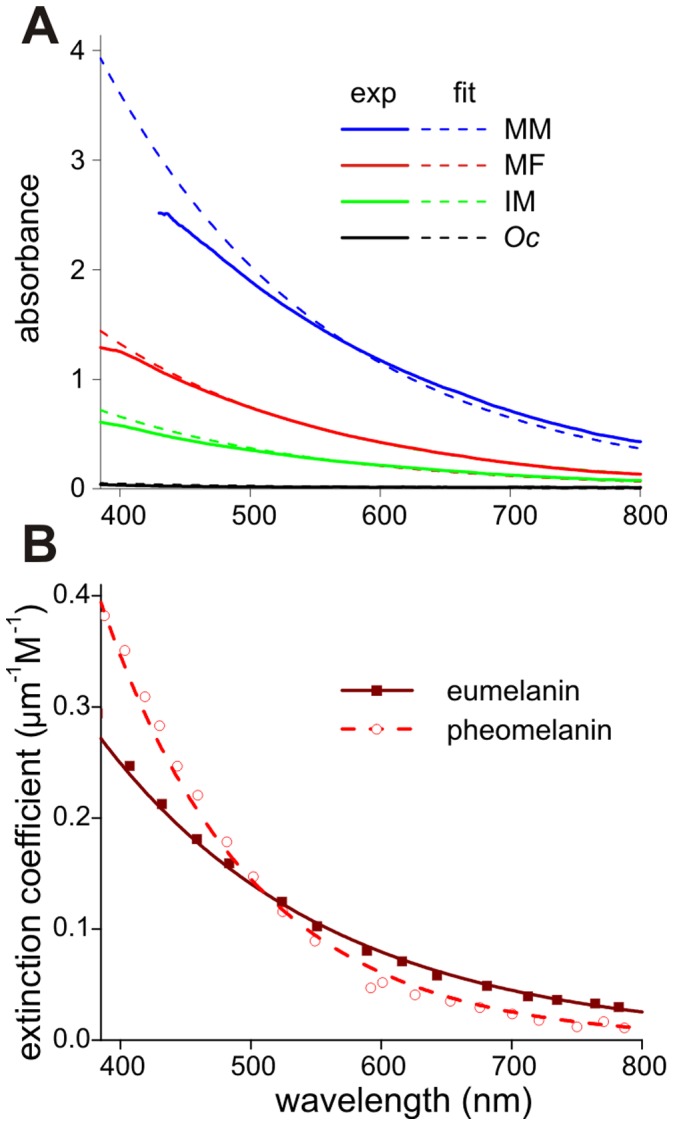
Absorbance spectra of the wings of **Figure 3** and of melanin. **A** Spectra derived from transmittance measurements with a microspectrophotometer measured from the wing membrane of the mature male (MM), mature female (MF), immature male (IM) damselfly and the dragonfly (*Oc*; continuous curves), fitted with an exponential function (dashed curves). **B** Extinction coefficient spectra of eumelanin and pheomelanin and exponential fits.

The measured transmittance spectra converted into absorbance spectra closely resemble melanin spectra (Figure 4A). To determine whether eumelanin or pheomelanin, the two common forms of melanin, was present in the damselfly wings, we used the extinction coefficients of both melanin forms, tabulated at http://omlc.ogi.edu/spectra/melanin/extcoeff.html (derived from [19]; Figure 4B). In the visible wavelength range, the melanin absorbance spectra approximate exponential functions [20]–[21]. We fitted the spectra of eumelanin and pheomelanin with the exponential function *ε* = *ε*_0_exp(-*λ*/*λ*_m_), and derived for eumelanin *ε*_0_ = 2.45 µm^−1^M^−1^ (1 M = 1 mol L^−1^) and *λ*_m_ = 175 nm, and for pheomelanin *ε*_0_ = 11.2 µm ^−1^M^−1^ and *λ*_m_ = 115 nm (Figure 4B). Exponential fits to the experimental absorbance spectra (Figure 4A) yielded *λ*_m_-values that well approximated the eumelanin value but strongly differed from that of pheomelanin. The spectra of Figure 4A were fitted with the function *D* = *D*_0_exp(-*λ*/*λ*_m_), with *λ*_m_ = 175 nm, yielding *D*_o_ = 0.45 (*Oc*), 6.5 (IM), 13.0 (MF) and 35.5 (MM). Normalized to the mature male, the relative melanin density of the various wings is 0.013 (*Oc*), 0.18 (IM), 0.37 (MF) and 1.0 (MM). In other words, the pigment density of the transparent dragonfly wings is no more than about 1% of that in the mature male damselfly. The fitted curves deviate slightly from the experimental spectra, notably at the shorter wavelengths, which is due to measurement errors occurring at high values of the melanin absorbance.

### The Complex Refractive Index of Damselfly Wings

**Figure 5:**
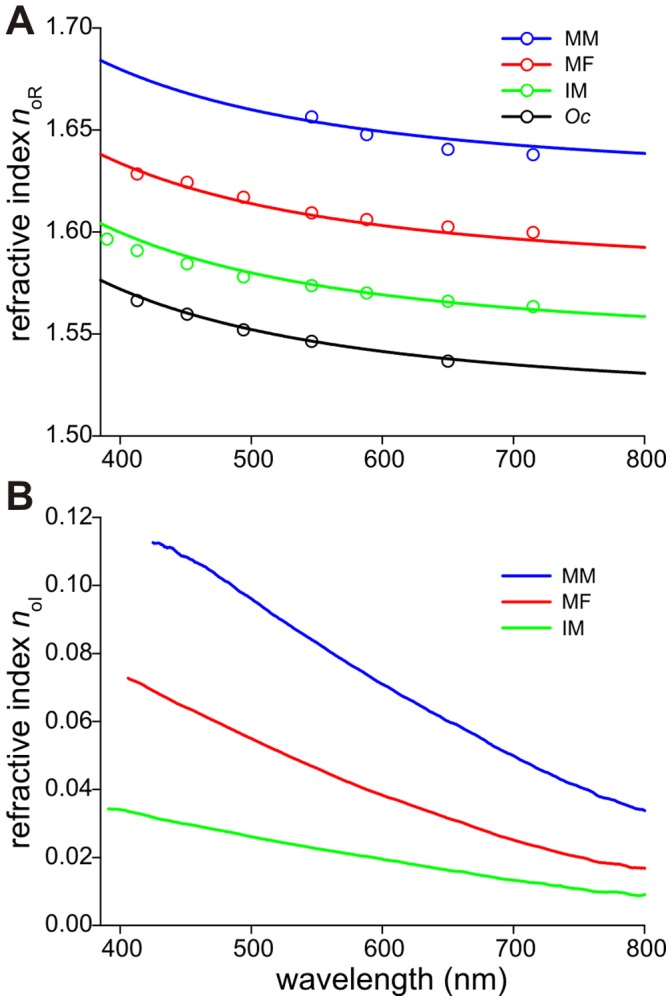
Wing refractive index as a function of wavelength for the damselfly *Calopteryx japonica* and the Black-tailed Skimmer dragonfly, *Orthetrum cancellatum*. **A** Real part of the refractive index, *n*_oR_, of the wings derived from the Jamin-Lebedeff measurements (Figure 6B) for the mature male (MM), mature female (MF), immature male (IM) and the dragonfly (*Oc*) (symbols). The black curve represents the chitin dispersion function derived from the glass scale of the butterfly *Graphium sarpedon* [22]. The other curves are obtained by adding a constant proportional to the melanin concentration derived from the absorbance spectra (Figure 4A). **B** Imaginary part of the refractive index, *n*_oI_, of the wings of three damselfly cases, derived from the absorbance curves together with estimates of the wing membrane thickness.

To determine the dependence of the refractive index on melanin density, we performed Jamin-Lebedeff interference microscopy on wing membranes of immature and mature male damselflies and of the dragonfly ***Orthetrum cancellatum*** (Figure 3, Supplementary Figures S2, S3). This yielded the real part of the refractive index, *n*_oR_(*λ*), of the four wings (Figure 5A, symbols) as well as the thickness of the wing membranes, being *d* = 1.30±0.03 µm (IM), 1.17±0.03 µm (MF), 1.83±0.04 µm (MM) and 2.50±0.05 µm (*Oc*). Together with the absorbance data, *D*(*λ*), of the three damselfly wings (Figure 4A), the imaginary refractive index (Figure 5B) was obtained from *n*_oI_(*λ*) = *D*(*λ*)*λ*/(4π*d*log_10_ *e*) (Eq. 3).

The dragonfly wing membrane is virtually completely transparent. The refractive index is then fully due to chitin, the basic material of insect wings. In a previous paper [22] we studied the chitin of the glass scales of the butterfly *Graphium sarpedon* and thus found that the refractive index of chitin is well described by the Cauchy dispersion equation, *n*_c_(*λ*) = *A*+*B*/*λ* ^2^, with *A* = 1.517 and *B* = 8.80·10^3^ nm^2^. The Cauchy equation with the same *A* and *B* values fitted the *n*_oR_(*λ*) values obtained for the transparent wings of the dragonfly remarkably well (Figure 5A). The n_oR_(λ) data points of the three damselfly wings were distinctly higher than those for the transparent dragonfly wing, as expected from the higher absorbances (Figure 4A). The curves through the data points were obtained as follows. Using Eq. 2, the concentration of melanin, *C* = *D*_o_/(*ε*_o_ *d*), was calculated with *ε*_0_ = 2.45 µm^−1^M^−1^ derived from the eumelanin fit and the values of D_o_ and d found for the various damselfly cases, yielding *C* = 2.0 M (IM), 4.5 M (MF) and 7.9 M (MM). Normalized to the mature male wing the concentration ratio factor then is 0.26 (IM), 0.57 (MF) and 1 (MM). The real part of the refractive index due to melanin must be similarly proportional to the concentration: *n*_mel_ = *ηC*. Using the three derived melanin concentrations, the function *n*_oR_(*λ*) = *n*_c_(*λ*)+*n*_mel_ = *n*_c_(*λ*)+*ηC*, with η = 1.35·10^−2^ M^−1^, indeed yields a satisfactory fit to the measured data points of the three wing cases (Figure 5A).

### Modelling the Reflectance Spectrum of the Blue Male Wing Veins

**Figure 6:**
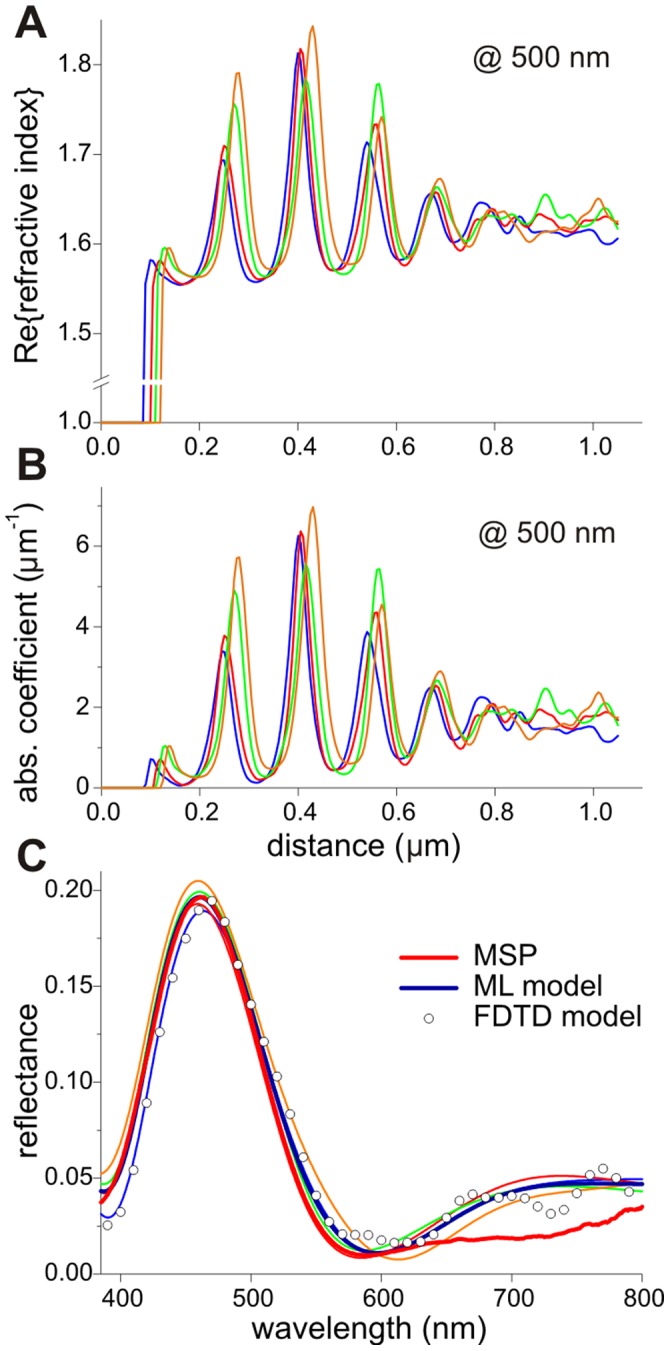
Refractive index and absorption coefficient profiles and resulting reflectance spectra of the male damselfly wing vein. **A** Real part of the refractive index as a function of depth, at wavelength 500 nm, following from the density profiles of the four lanes in the boxed area of Figure 2A. **B** Absorption coefficient corresponding to the refractive index profile of **A**. **C** Reflectance spectrum measured with a microspectrophotometer (red curve, MSP, diameter of the measurement area ∼15 µm) of a male wing vein and spectra calculated with a multilayer model. The thin curves were obtained with the refractive index profiles of the four lanes of Figure 2A, and the blue bold curve (ML model) is the average. The symbols represent a reflectance spectrum calculated with a FDTD model that used refractive index values derived from the densities in the full boxed area of Figure 2A.

The derived refractive index values allow us to investigate the blue reflections of the veins of male wings. Applying about normal illumination, the reflectance spectrum of the wing veins of male *Calopteryx japonica* damselflies measured with a microspectrophotometer (MSP, Figure 6C), has a distinct peak in the blue, alike the spectrum measured from the complete wing with an integrating sphere (Figure 1C). This spectrum must be caused by the melanin-chitin multilayer in the wing veins. To model the reflectance with multilayer theory, we first analyzed the density of the transmission electron micrograph of the vein of Figure 2A in four lanes (width 500 nm, length 1050 nm) perpendicular to the vein surface. In each lane the average density of a 5 nm thick layer was determined, yielding 210 density values. The minimum density in the vein was assumed to be due to chitin, and the additional density was assumed to be linearly proportional to the concentration of melanin, thus yielding the dependence of the concentration on the location *x*, *C*(*x*).

Using the relations derived above, *n*_oR_(*λ*) = *n*_c_(*λ*)+*ηC* and *n*_oI_(*λ*) = *C λ ε*_0_(*λ*)exp[−λ/λ_m_]/(4*π*log_10_(*e*)), we calculated the refractive index profiles, *n*_o_(*x*) in the four lanes. We then implemented the resulting refractive index values in a multilayer model [6] and calculated the reflectance spectrum for normal illumination. We scaled the refractive index profiles (both the real and complex part) until the modelled reflectance spectra fitted the experimental reflectance spectrum. A peak wavelength of 460±2 nm, well corresponding with the experimental peak wavelength, followed with the refractive index profiles of Figure 6A,B, where the peak value of the real part of the refractive index is about 1.8. Of course, a distinctly lower value follows for the average refractive index of the melanin-chitin multilayer: ∼1.65.

We have to note here that the deduced refractive index values assume negligible deformations of the dry and solid wing veins during processing for TEM. Furthermore, the shape of reflectance spectra can be reliably measured by microspectrophotometry, but calibration of its amplitude is cumbersome due to several geometrical factors, for instance the curved shape of the veins. The spectra of Figure 6C show a peak reflectance of ∼0.2. This value results from the multilayer model calculations for normal illumination. The experimental spectrum was adjusted to this peak reflectance.

## Discussion

The male damselfly *Calopteryx japonica* has dark-blue wings due to multilayered wing veins, which act as interference reflectors. The wing area taken up by the veins is small (Figure 1A) and the blue reflectance is low (Figure 1C), but the blue colour is nevertheless prominently visible because of the very low reflectance of the wing membranes (Figure 1A), which is due to the high melanin concentration. The female has brown wings, because the melanin concentration in the female wing is lower than in the male wing, and the female wing veins have no melanin-chitin multilayers.

The damselfly wing membranes approximate thin plates, which allowed measurement of the melanin refractive index as a function of wavelength. The data implemented in a classical multilayer model, and using the anatomy of the layered wing veins, yielded a blue-peaking reflectance spectrum matching experimental spectra surprisingly well. The calculated spectra were obtained by considering small parts of the vein structure as being infinite multilayers, which may well be questioned. A more rigorous finite-difference time-domain (FDTD) model for a large part of the vein cuticle, where the cross-section of Figure 2 was binarized and refractive index values of Figure 5 were assigned, produced a very similar spectrum (Figure 6C, symbols; in preparation).

By using the differently pigmented wings of the immature and mature damselflies, *Calopteryx japonica*, we found that the refractive index increases by a constant, approximately wavelength-independent amount, proportional to the melanin concentration. The real part of the refractive index at 500 nm for the different cases studied is 1.552 (chitin: *Oc*), 1.580 (IM), 1.615 (MF), and 1.663 (MM). The wavelength-dependent decrease of the refractive index, for the mature male wing from 1.683 at 400 to 1.646 at 700 nm (Figure 5a), results from the dispersion of the chitin.

The refractive index of melanin has remained uncertain for several decades and thus was haphazardly assumed to be 2.0 (e.g. [7] [14]), while the effect of its strong absorption at short wavelengths has been commonly neglected in modelling studies. Here we have found that the real part of the melanin refractive index is well below 2.0 and our calculations showed that absorption plays a major role in shaping reflectance spectra and visibility. The obtained data will be useful for further studies aimed at understanding animal coloration and for optical studies on melanised tissues.

## Supporting Information

Figure S1 **A wing of a mature male** ***Calopteryx japonica*** **, of which the right half was put for three hours in a solution of warm 3% H_2_O_2_, resulting in bleaching of the melanin pigment. Bar: 0.5 cm.**
(TIF)

Figure S2
**Jamin-Lebedeff microscopy of a damselfly wing piece and measured intensity curves.** **A–C** Photographs of a wing piece of a mature female immersed in a fluid with refractive index 1.56 (at 588 nm) using 546 nm light and angular position of the analyzer −70° (**A**), −10° (**B**) and+50° (**C**). The arrow heads in **a** indicate wing veins. The circles in **B** indicate the reference area (r) and object area (o) where the light intensity was evaluated. Bar: 50 µm. **D** Normalized intensity evaluated at the reference area (ref) and the relative intensity of the object area (obj) for wavelengths 451 and 650 nm. The data points were fitted with the sinusoidal function 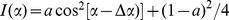, where *a* is the amplitude, *α* the angular position of the analyzer, and Δ*α* the phase shift.(TIF)

Figure S3
**Amplitude and phase shift of the sinusoidal fits to the analyzer-dependent intensity curves measured for a wing piece of a mature female damselfly.** **A** Amplitudes for three immersion fluids with refractive index (at 588 nm) 1.56, 160 and 1.64, together with *T* ^½^, the square root of the transmittance spectrum, measured microspectrophotometrically. **B** Angular phase shift measured for the three immersion fluids (indicated by arrows) and various wavelengths. The data points were fitted with the linear function 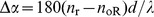, where *n*_r_ is the refractive index of the reference medium, *n*_oR_ is the real part of the refractive index of the object, *d* is the thickness of the object, and *λ* the wavelength.(TIF)
