# Supplementary figures and images for: Sexual Dichromatism of the Damselfly Calopteryx japonica Caused by a Melanin-Chitin Multilayer in the Male Wing Veins
Source: PLoS One. 2012 Nov 20;7(11):e49743. doi: 10.1371/journal.pone.0049743 (PMC3502265; doi:10.1371/journal.pone.0049743)

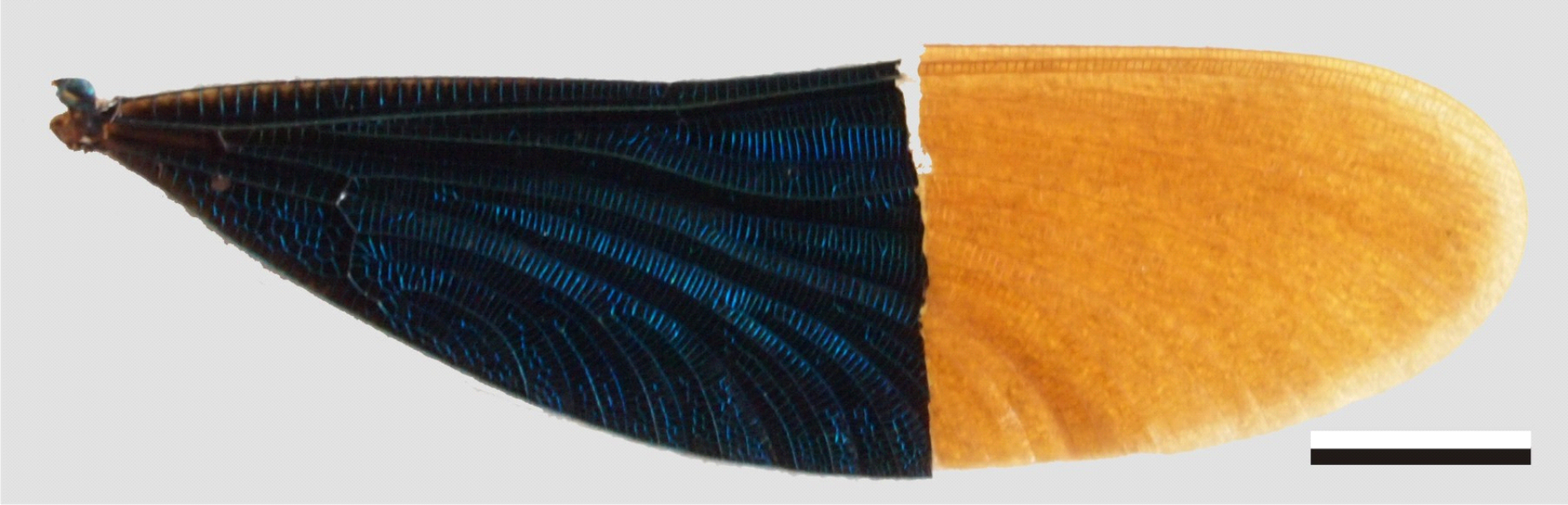

Supplement: Figure S1 — A wing of a mature male Calopteryx japonica , of which the right half was put for three hours in a solution of warm 3% H2O2, resulting in bleaching of the melanin pigment. Bar: 0.5 cm. (TIF) [file pone.0049743.s001.tif]

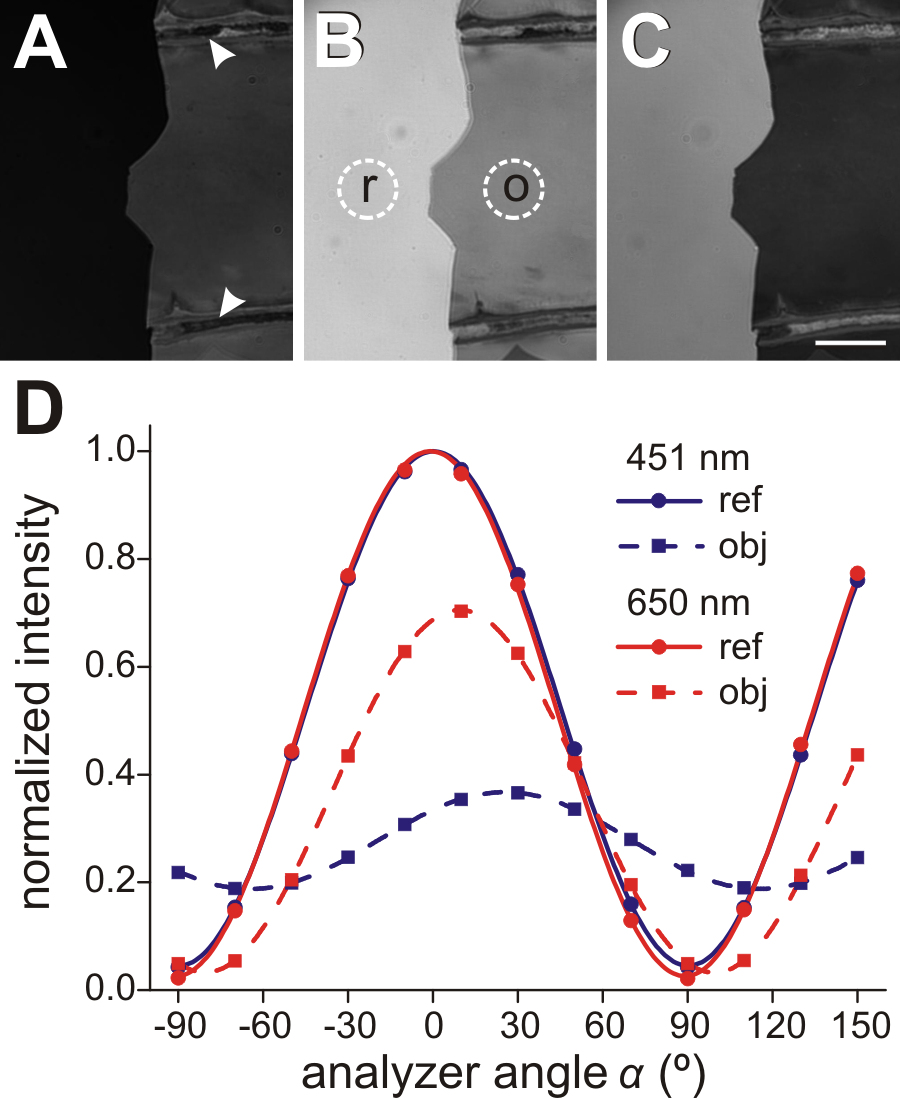

Supplement: Figure S2 — Jamin-Lebedeff microscopy of a damselfly wing piece and measured intensity curves. A–C Photographs of a wing piece of a mature female immersed in a fluid with refractive index 1.56 (at 588 nm) using 546 nm light and angular position of the analyzer −70° (A), −10° (B) and+50° (C). The arrow heads in a indicate wing veins. The circles in B indicate the reference area (r) and object area (o) where the light intensity was evaluated. Bar: 50 µm. D Normalized intensity evaluated at the reference area (ref) and the relative intensity of the object area (obj) for wavelengths 451 and 650 nm. The data points were fitted with the sinusoidal function , where a is the amplitude, α the angular position of the analyzer, and Δα the phase shift. (TIF) [file pone.0049743.s002.tif]

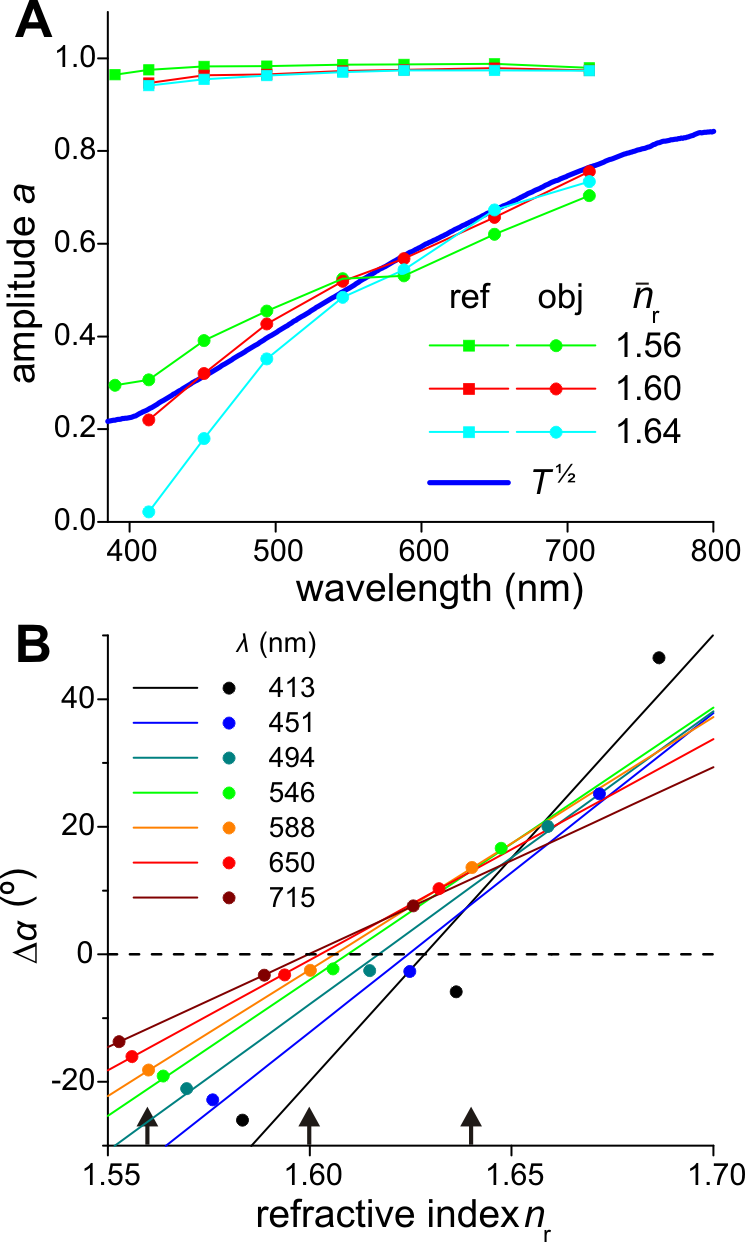

Supplement: Figure S3 — Amplitude and phase shift of the sinusoidal fits to the analyzer-dependent intensity curves measured for a wing piece of a mature female damselfly. A Amplitudes for three immersion fluids with refractive index (at 588 nm) 1.56, 160 and 1.64, together with T ½, the square root of the transmittance spectrum, measured microspectrophotometrically. B Angular phase shift measured for the three immersion fluids (indicated by arrows) and various wavelengths. The data points were fitted with the linear function , where n r is the refractive index of the reference medium, n oR is the real part of the refractive index of the object, d is the thickness of the object, and λ the wavelength. (TIF) [file pone.0049743.s003.tif]
